# Acute Motor Axonal Neuropathy in Lupus Nephritis

**DOI:** 10.7759/cureus.55603

**Published:** 2024-03-05

**Authors:** Diljeet Bodra, Amith Vijay Leon D'Souza, Essar Khan

**Affiliations:** 1 Nephrology, Father Muller Medical College and Hospital, Mangalore, IND

**Keywords:** systematic lupus erythematosus, acute motor axonal neuropathy (aman), lymph nodes, sle and lupus nephritis, nerve conduction testing

## Abstract

A prevalent clinical scenario is provided in this case study, in which a 22-year-old lady with a five-year history of lupus nephritis with acute motor axonal neuropathy presents for therapy. The patient received immunomodulator medication and steroids to control her symptoms to keep up with her everyday life despite the absence of comorbidities such as hypertension, diabetes, and hypothyroidism. No laboratory measures were changed, including hemoglobin, serum creatinine, or thyroid function. Examining the nervous system indicated a potentially harmful consequence, underscoring the significance of prompt investigation and treatment. This research highlighted the importance of attention in cases with lupus nephritis, showing how early medical care can prevent serious neurological problems and contribute to the patient's general well-being.

## Introduction

Lupus nephritis is a type of glomerulonephritis that represents a significant expression of organ involvement in the autoimmune disorder known as systemic lupus erythematosus (SLE). Lupus nephritis sometimes serves as the initial clinical symptom leading to the diagnosis of SLE [1]. Based on previous scholarly research conducted by Chasset et al., it has been proposed that lupus-like illness may develop due to various factors, including infections, hematological malignancies, solid tumors, and similar causes [2].

However, the occurrence of renal involvement in the form of lupus nephritis is few, with a reported prevalence ranging from 3% to 7% [3]. This study presents a unique instance of coexisting proliferative and membranous lupus nephritis, which was shown to be related to mantle cell lymphoma. Furthermore, it is observed that the patient experienced partial renal recovery subsequent to chemotherapy treatment. The nervous system has emerged as a prominent target in the context of SLE, with a substantial proportion of patients exhibiting established involvement of the nervous system [4].

A recent study examining individuals diagnosed with SLE revealed that 36.54% exhibited neurological symptoms [5]. The most prevalent among these manifestations was cognitive impairment, affecting 57.89% of patients and presenting as issues with memory, attention, and cognitive skills [6]. Seizure conditions were identified in 42.1% of patients, indicating a heightened susceptibility to seizures in individuals with SLE [7]. Less common but still present in the cohort were psychosis, motor disorders, and aseptic meningitis [8]. Psychosis, characterized by a lack of contact with reality, and motor disorders, involving abnormal movements or postures, were observed, albeit less frequently [9]. Aseptic meningitis, the inflammation of the meninges without a bacterial infection, represented another uncommon neurological consequence in individuals with SLE [10]. This comprehensive understanding of diverse neurological manifestations in SLE underscores the need for multifaceted approaches in both diagnosis and management. In a related case report, an individual developed Guillain-Barré syndrome (GBS) as a result of SLE, with paralysis manifesting suddenly [11]. GBS is a subtype known as acute motor axonal neuropathy (AMAN), where motor nerve fibers are destroyed, sparing sensory nerve fibers [12]. Despite conventional therapeutic approaches, such as plasmapheresis and immunomodulator therapy, the case study observed that SLE manifesting as GBS did not exhibit a favorable response to these interventions [13]. This emphasizes the intricate nature of autoimmune illnesses and underscores the necessity for personalized treatment strategies (immunosuppressive therapy selection, adjunctive therapies, and aggressive induction therapy). Enhancing the therapy of SLE requires a comprehensive exploration and understanding of its varied neurological symptoms [14].

## Case presentation

The patient, a 22-year-old female, arrived at our department with a primary concern of experiencing trouble transitioning from a supine position to a sitting posture. This symptom has been ongoing for two weeks. Significantly, she indicated the absence of any additional comorbidities and refuted any recent occurrences of fever or diarrhea. The patient's medical records indicated the presence of lupus nephritis, which was definitively identified in 2017 by renal biopsy. The severity of the condition was classified as International Society of Nephrology (ISN) grade 3+/-4. Since the initial diagnosis, she has been receiving ongoing care through the administration of immunomodulator therapy and steroid medicines. Notwithstanding her persistent ailment, she expressed being asymptomatic and fully involved in her daily pursuits. Upon evaluation, she was advised to receive Solu-Medrol intravenously for three days, followed by a tapering course of steroids over one month, in addition to immunomodulators as planned. Within three days, she demonstrated gradual improvement, leading to her discharge with regular follow-up. Collaboration with the neurology and anesthesia teams would be necessary if respiratory depression occurs.

The laboratory findings revealed a hemoglobin (Hgb) level of 10 g/dL, a serum creatinine level of 0.5 mg/dL, and the presence of 2+ protein in the urine routine/microscopy analysis. Serum levels of vitamin B12 were 250 pg/mL, thyroid-stimulating hormone (TSH) was 0.5 mIU/L, blood potassium concentration was (3.5 mEq/L), and serum creatine phosphokinase (CPK) concentration was 20 g/L; these were all considered to be significant laboratory values.

The neurological assessment was performed to clarify the underlying cause of the patient's challenge in adopting a seated position. Unfortunately, the document lacks specific details regarding the neurological observations. A thorough neurological examination generally encompasses the evaluation of cognitive function, cranial nerve integrity, motor abilities, reflexes, sensory perception, coordination, and gait. Due to the intricate nature of the case and the presence of the patient's pre-existing autoimmune disorder, it is imperative to conduct a comprehensive assessment of the nervous system to detect any possible neurological manifestations or problems.

The scenario described highlights the necessity of adopting a multidisciplinary strategy that incorporates the expertise of rheumatologists, nephrologists, and neurologists to comprehensively address the patient's condition. Based on the results of the neurological examination, it may be necessary to conduct further investigations, such as imaging scans or other neurological testing. The timely coordination and synthesis of research findings across several disciplines are crucial to delivering a comprehensive and efficacious treatment strategy that has been specifically customized to the individual patient's distinct medical conditions.

Table 1 presents the clinical examinations of the hips, focusing on the range of motion assessed for both the right and left sides.

**Table 1:** The clinical examinations of the hips, knees, and ankle, focusing on the range of motion assessed for both the right and left sides

Range of motion	Right	Left
Hips		
Flexion	3/5	3/5
Extension	4/5	4/5
Knees		
Flexion	4/5	4/5
Extension	5/5	5/5
Ankles		
Flexion	4/5	4/5
Extension	5/5	5/5

## Discussion

SLE is an autoimmune disorder that manifests with the involvement of several organs, diverse clinical symptoms, and varying levels of severity. Among these manifestations, lupus nephritis stands out as a prevalent contributor to both morbidity and mortality. However, earlier studies have shown that a variety of variables, including infections, hematological malignancies, solid tumors, pharmacological agents, and other comparable reasons, can result in “SLE.” Furthermore, it has been noted that renal problems are often linked to SLE [15].

In this report, we provided a case study of a 22-year-old female patient who has reported experiencing difficulty transitioning from a supine position to a sitting posture for the past two weeks. The noteworthy aspect of this case was the absence of comorbidities and the patient's overall well-being in their everyday activities despite the chronic nature of membranous lupus nephritis. The laboratory results consist of a Hgb level of 10 g/dL, a serum creatinine level of 0.5 mg/dL, and a 2+ protein in the urine macroscopy.

In a study by Tzioufas et al., the findings revealed three distinct groups among individuals diagnosed with SLE, categorized based on their Hgb levels. Group A comprised 45 patients with Hgb levels exceeding 12 g/dL, group B included 26 patients with Hgb levels ranging from 10.1 to 12 g/dL, and group C consisted of 21 patients with Hgb levels equal to or below 10 g/dL. To assess disease activity in SLE patients, the researchers employed "The European Consensus Lupus Activity Measurement Scale" [16].

In this case study, a comprehensive evaluation of the patient's health includes key parameters such as TSH level, serum vitamin B12 level, serum potassium level, and CPK level, providing insights into both endocrine and musculoskeletal aspects. While the neurological examination lacks a detailed description, it encompasses tests of hip flexor and extensor muscle strength. The observed 3/5 strength symmetrically on both sides for flexion and 4/5 strength for extension suggests a moderate degree of muscular weakening, possibly contributing to the reported difficulty in assuming a sitting posture. Recognizing the complexity of the issue, a collaborative approach involving nephrologists, neurologists, and rheumatologists is essential.

In 2020, Aktaş conducted a study, performing comparative analyses across various demographic factors, including age, gender, TSH, free-T4, vitamin D, and anti-thyroid peroxidase (TPO). The study also delved into exploring the relationship between anti-TPO levels and vitamin B12 within these distinct vitamin B12 level groups [17]. These findings contribute valuable insights into the intricate associations between anti-TPO antibodies and vitamin B12 and D levels in the context of autoimmune hypothyroidism, shedding light on potential factors influencing the condition and providing a basis for further exploration and clinical considerations.

The purpose of the neurological examination was to determine the real cause of the stated disability. However, a complete understanding of the situation is hampered by a lack of specific information regarding neurological observations. In order to detect any possible neurological manifestations or consequences related to lupus nephritis or its therapy, it is necessary to conduct a comprehensive neurological assessment; it includes an assessment of the cranial nerves, motor function, reflexes, coordination, mental state, and gait.

## Conclusions

In conclusion, the intricate case of the 22-year-old female patient with lupus nephritis emphasizes the need for a nuanced and collaborative medical approach. Despite the persistence of a challenging symptom, the patient's overall well-being has been maintained through diligent care, incorporating immunomodulator therapy and steroids. The laboratory findings, notably an Hgb level of 10 g/dL and the presence of proteinuria, are indicative of membranous (class V) lupus nephritis, a unique nephrotic variant among lupus nephritis subtypes. This distinction emphasizes the complexity of autoimmune disorders and their diverse presentations. Furthermore, the case underscores the significance of a detailed neurological assessment, highlighting the complexity of symptoms arising from the patient's autoimmune disorder. As the difficulty in transitioning from a supine to a sitting position persists, it becomes imperative to prioritize a thorough evaluation that goes beyond routine examinations. This necessitates a holistic, multidisciplinary approach involving rheumatologists, nephrologists, and neurologists. Such collaborative efforts are paramount in not only understanding but effectively managing the multifaceted challenges posed by autoimmune disorders, ensuring comprehensive and personalized care tailored to the unique needs of the patient.
